# Human rights and nutritional care in nurse education: lessons learned

**DOI:** 10.1177/09697330211057226

**Published:** 2022-02-07

**Authors:** Elisabeth Irene Karlsen Dogan, Laura Terragni, Anne Raustøl

**Affiliations:** 87368 VID Specialized University, Oslo, Norway; Faculty of Health Sciences, 158935 Oslo Metropolitan University, Oslo, Norway; Faculty of Health Studies, 87368 VID Specialized University, Oslo, Norway

**Keywords:** human rights, nurse education, ethical competence, nutritional care, right to food and older adults

## Abstract

**Background:** Food is an important part of nursing care and recognized as a basic need and a human right. Nutritional care for older adults in institutions represents a particularly important area to address in nursing education and practice, as the right to food can be at risk and health personnel experience ethical challenges related to food and nutrition. **Objective:** The present study investigates the development of coursework on nutritional care with a human rights perspective in a nursing programme for first-year nursing students and draws upon reflections and lessons learned. **Research design:** The study utilized educational design research. The coursework, developed through two rounds, combined on-campus learning and clinical placement in nursing homes. Nursing studentsʼ perspectives and experiences gathered through focus groups and a written assignment informed the development and evaluation of the coursework. **Participants and research context:** In the first round, multistage focus group interviews were conducted with 18 nursing students before, during and after placement. In the second round, four focus group interviews with 26 nursing students were conducted shortly after placement. **Ethical consideration:** The study was approved by the Norwegian Centre for Research Data. **Findings:** Three main ʽlessons learnedʼ emerged regarding introducing a human rights perspective in nursing education: 1) the contribution of the human rights perspective in changing the narrative of ʽvulnerable and malnourished patientsʼ, 2) the importance of relationships and experiences for learning about human rights and 3) the benefit of combining development of ethical competence with a human rights perspective. **Conclusion:** A human rights perspective enabled the students to give meaning to nutritional care beyond understanding of food as a basic physical need. Incorporating human rights in nursing education can support nursing students and nurses in recognizing and addressing ethical and structural challenges and being able to fulfil the right to food for patients.

## Introduction

Nutritional care for older adults in nursing homes represents a particularly important area to address in nursing education and practice. In Norway, several concerns have been raised in this regard: an overly long overnight fast,^1^ little or no influence on food choices,^2^ mealtimes being scheduled in accordance with staff work schedules rather than residents’ needs,^3^ inadequate documentation of nutritional treatment,^4^ and underrecognizing and undertreatment of patients nutritionally at risk.^5^ Health personnel caring for older adults experience ethical challenges related to food and nutrition on a daily basis.^6,7^ These include dealing with persons at risk of malnourishment who refuse to eat, persons with dementia who are unable to express their needs, arguing among residents during mealtimes, or lack of proper working conditions for healthcare staff to support residents during mealtimes.^6,7^

Nutritional care is an important part of nursing.^8^ In this field, eating and drinking are traditionally conceived as a physical need.^8,9^ and the moral, social, cultural, psychological and spiritual aspects of eating and drinking are often disregarded.^10^ Studies indicate that nurses and nursing students lack nutrition-related knowledge,^11-13^ and few studies have discussed pedagogic methods in nutrition education in nursing.^14,15^ The alarming number of older adults in institutions suffering from malnutrition may indicate that the current approach to food and nutrition is inadequate and that a shift in the approach to nutrition in nursing education may be needed. A human rights perspective has received increased attention in recent years in nursing practice and care.^16,17^ There is however limited knowledge about how a human rights perspective have been introduced in nursing education regarding nutritional care. This article discusses lessons learned from a study investigating the introduction of food and nutrition as a human right in nursing education.

## Background

Human rights are the rights one has because one is a human being,^18^ while human dignity is generally acknowledged as the source of human rights.^19^ There is broad consensus that human rights rest on a life of dignity and thus constitute a broader concept than just human survival and basic needs thinking.^19^

The human right to food has been recognized since the Universal Declaration of Human Rights was adopted by the UN General Assembly in 1948^20^ and was later recognized by international law.^21,22^ The right to food has been officially interpreted by the UN Committee on Economic, Social and Cultural Rights (CESCR),^23^ who specify that this right is of crucial importance for the enjoyment of all rights – and that it is not limited to calories and nutrients, but incorporates human dignity, dietary needs, food safety and food security. The right to food has been defined by the first United Nations’ Special Rapporteur on the right to food as:

The right to have regular, permanent and unrestricted access, either directly or by means of financial purchases, to quantitatively and qualitatively adequate and sufficient food corresponding to the cultural traditions of the people to which the consumer belongs, and which ensure a physical and mental, individual and collective, fulfilling and dignified life free of fear.^24^

The right to food, like any other human right, implies three types or levels of obligations for States parties: the obligations to *respect*, *protect* and *fulfil or facilitate.*^23^ Norway became a State party to the International Covenant on Economic, Social and Cultural Rights (ICESCR) in 1972, by way of ratification.^25^ Nevertheless, in recent years, concerns have been raised regarding the violation of this human right in Norwegian healthcare services.^26-28^ While the state of Norway has the overall obligation to make responsible policies around caring for older people, various public actors have the immediate duty of carrying these out.^29^ When someone is entitled to a right, someone else has the duty to fulfil this right. The concept of ‘duty bearers’ is used to identify the nested rings of responsibilities towards securing this right. Each ring of duty bearers should support and give guidance to the duty bearers closest to the right holders.^29^ In the case of older adults in institutions, the health staff are the duty bearers closest to the nursing home residents and should be supported in their efforts to fulfil residents’ rights by the administration and management.^29^

To promote the right to food as well as other fundamental rights, UN agencies have proposed a human rights-based approach (HRBA).^30^ In an HRBA, the processes towards a desired outcome are guided by a set of principles including human dignity; equality and non-discrimination; participation and inclusion; empowerment; accountability; transparency; and respect for the rule of law.^30^ An HRBA is a bottom-up approach, where the above-mentioned principles can be operationalized and put into everyday practice.^31^

Human rights education (HRE) has expanded in recent decades, across academic disciplines.^32^ However, calls remain for HRE to be integrated into training for health personnel.^32,33^ In the field of nursing, recent research highlights the need for improving nursing students’ human rights sensitivity,^34,35^ and for studies on how nursing students evaluate human rights content in courses.^36^

The present study, therefore, investigates the introduction of food and nutrition as a human right in a nursing programme and whether a human rights perspective may support nursing students facing ethical challenges related to their professional work. This article is part of a larger study on the development of coursework about the right to food and nutrition for nursing students: while this article draws upon reflections and lessons learned in developing the coursework, previous articles from the study investigated students’ understanding of and learning about the right to food.^37,38^

## Incorporating students’ perspectives and learning through their interactions, relationships and experiences

### Theoretical approach: Human rights education for nursing students

The study was framed around HRE as it is described by Tibbitts.^39^ The aim of HRE in our context was to teach nursing students how to advocate for social justice and act on behalf of patients whose rights are neglected.^39^ HRE involves the following components^40^: First, HRE must include both content and processes to teaching human rights^40^; second, it must include goals related to the content, value, skills and action-oriented components of HRE.^39^ The main objectives of HRE can be described as to educate *about* human rights, to educate *through* human rights and to educate *for* human rights.^41,42^ To educate through human rights aims to change values and behaviours of an institution and to educate through human rights aims to empower for social justice.^17^ HRE means thus providing not only the knowledge about human rights and the mechanisms that protect them but also the skills needed to promote, defend and apply human rights in daily life.^40^ Moreover, emphasis is placed on the importance of learning through socialization^39^ and on making the abstract content of human rights policies both contextualized and relevant for learners.^43^

### Development of the coursework: Educational design research

An educational design research (EDR) approach was utilized to develop the coursework.^44^ EDR embraces practical research methodology, bridging research and practice in formal education^44^; this approach emphasizes situations in real learning settings and stresses the importance of improving practice in specific contexts. EDR includes three main phases, which are both iterative and flexible: 1) analyses and exploration, 2) design and construction and 3) evaluation and reflection.^44^ In the study, the coursework on the right to food was developed through two iterative rounds, with some modification of the coursework between the two rounds which took place in 2017 and 2018. The coursework consisted of a 2-day module at campus that was part of a 7-week course on older adults in care facilities, followed by a 7-week clinical placement at a nursing. This coursework took place in the students’ second semester, right before and during their first clinical placement. One of the learning outcomes related to human rights was ‘The student can apply knowledge of human rights principles associated with the right to adequate food and initiate measures connected to nutrition, food and meals’.

**Figure 1.**
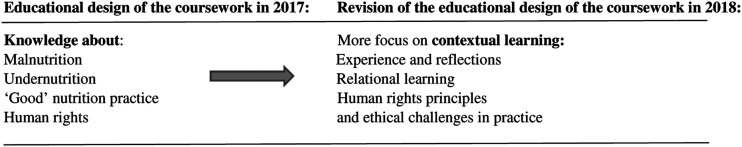
Evolution of the module.

In the first round of the coursework (2017), two teachers (one of whom is the first author) developed the 2-day, on-campus module. Two multistage focus group interviews were conducted with 18 nursing students before, during and after placement to obtain knowledge and understanding about nursing students’ views on the right to food throughout their placement.^37^ Based on the findings, changes were made to the initial coursework. Those findings underscored the importance of clinical placement for nursing students with regard to learning about human rights. One of the main findings was that students learned about human rights when they experienced challenges in context and gained contextualized understanding.^37^ The students’ perspectives and experiences about the right to food helped shift the coursework towards facilitating learning through their relationships and experiences (see Figure 1).

Topics that the students found challenging in the first round^37^ were emphasized in the second round of the coursework: namely, ethical challenges related to food, nutrition, diet and meal situations; structural challenges related to nutritional care in practice; roles in nutritional care; handling challenges around the right to food in nursing homes; empowering students to reflect on and discuss ethical challenges and rights violations with their supervisor and fellow students and contextualizing learning about the right to food using the students’ own experiences during their placement (see Table 1). Further emphasis was placed on human rights principles – dignity, empowerment, accountability, participation and non-discrimination – in relation to food and mealtimes through discussions and reflections in small groups, aiming at making the students more aware of the complexity of nutritional care. This was an effort to strengthen the coherence between campus and placement learning and to promote a human rights perspective in practice.

**Table 1.** Themes on-campus lectures and written assignments in placement.

Campus	Placement
1. The importance of the right to food and meals in the institution 2. Human rights principles in nursing homes 3. Malnutrition, undernutrition 4. Food care for dementia, heart and vascular diseases, stroke	1. Create and register a 24-h meal plan for a resident, including nutrients, fluids, carbohydrates, proteins and fats, iron and vitamin D. Write a brief account of how you will care for and meet the resident’s needs if their nutritional intake is too low or high. Consider the residents’ nutritional intake in relation to their nutritional needs
5. Good nutrition practice	2. Make a brief assessment of the resident’s nutritional status using their BMI, nutritional needs and nutritional risk (MNA)
6. Cultural and religious aspects of food and meals	
**7. Ethical challenges related to food, nutrition, diet and meals**	**3. Is the resident taking medicine or do they have a disease where food and diet are important?**
**8. Structures in practice**	**4. Have you experienced ethically challenging situations related to food and meals? Which measures will you emphasize in line with ethical principles and the right to food as a human right?**
**9. Role in nutritional care**	
**10. Assignment in placement**	
	**Discuss and reflect with your placement supervisor and with a fellow student. See the literature about this topic**

*Note.* Changes between the two rounds are marked with **bold text**.

The lectures on campus were also centred around organizational structures in the clinical setting that can cause violations of the right to food; the students were given examples from Norwegian healthcare services, such as a lack of proper routines for mealtimes or the screening of undernutrition. Students were also introduced to the concept of dual loyalty (conflict between obligations to their patient and loyalty to their employer),^45^ through examples from the teachers and through small-group discussions. In addition, the students were introduced to the ‘Nurses and Human Rights’ statement from the International Council of Nurses for discussion.^45^ Finally, the written assignment for the coursework was amended to require students to describe and discuss an ethically challenging situation they had experienced during placement, and to encourage them to discuss this situation with their supervisor or fellow students. The mini nutritional assessment (MNA) was also carried over from 2017, following national guidelines. Students registered the 24-h documentation about the residents’ intake in a free computer programme that offered information about kcal intake, nutrients and fluid intake. However, in 2018, the students were encouraged to reflect upon these results in relation to ethical challenges and the right to food.

After making the changes outlined above, we evaluated the 2018 coursework.^38^ Focus group interviews with 26 nursing students were conducted following placement, and their written assignments were used to evaluate their learning about the right to food.

The findings from this second evaluation, together with the experiences from the first round of the coursework, provided relevant ‘lessons learned’. These were useful for meeting the main aims of our research related to 1) understanding how to introduce food and nutrition from a human rights perspective in a nursing programme and 2) whether this perspective could support nursing students facing ethical challenges related to their professional work.

### Ethical considerations

The study was approved by the Norwegian Centre for Research Data and carried out in accordance with the Declaration of Helsinki. The participants were given verbal and written details of the study’s purpose prior to participation, and we obtained informed written consent from all the participants. Since the first author also was one of the students’ university instructors, it was ensured and emphasized that what was said in focus group interviews would not affect the students’ evaluation. To protect patients’ confidentiality, participants were reminded before participating in the focus groups and contributing the assignments that no identifiable information about individual patients should be revealed.

## Discussion: Lessons learned

Three key ‘lessons’ were learned in introducing food and nutrition from a human rights perspective in a first-year nursing programme: 1) the contribution of the human rights perspective to changing the narrative of ‘vulnerable and malnourished patients’, 2) the importance of students’ learning about human rights through their relations and own experiences and not merely theoretically and 3) the importance of combining the development of ethical competence and a human rights perspective.

### Contribution of the human rights perspective to changing the narrative of ‘vulnerable and malnourished patients’ to a systems perspective of care for older adults

The first lesson learned was that the introduction of a human rights perspective helped the students deconstruct the narrative of ‘vulnerable and malnourished patients’ in nursing homes that dominates the literature^46,47^: it provided them with a ‘vocabulary’ for naming situations as violations and for identifying responsibilities. By focusing on food as a right and not merely on food as a physical need, students learned to counter this narrative revolving around ‘the problem’ of undernutrition as something that can be solved by inducing residents to gain weight, and by the provision of more energy-dense food. Findings from the present study indicate that HRE makes students aware that relying on this dominant narrative can be misleading, as it places under- and malnutrition as a problem of the individual patient or the individual nurse. This narrative may shift attention away from the complex dimensions of food – such as the ethical, cultural, social, psychological or religious aspects^48^ – and from the structural aspects related to nutritional care in nursing homes, pointing to healthcare staff as duty bearers and residents as right holders.^29,30^ Other studies also indicate that HRE can contribute to challenging dominant narratives and inviting the inclusion of new perspectives.^49^

Several studies stress the importance of developing a language about human rights.^16,50^ In the present study, when a human rights perspective was introduced, a human rights vocabulary seemed to empower the students to advocate for patients’ rights and to develop ethical competence.^38^ For instance, one of the students in the study justified her serving of food outside regular mealtimes by referring to the fact that food is a human right of the residents.^38^ The students also discussed the importance of respecting the residents’ wishes in meal situations – for example, with whom the residents shared a table during mealtimes – by referring to the importance of residents participating in decisions regarding their daily routines. These findings are supported by the Carnegie Foundation National Study of Nursing Education,^51^ which emphasized the importance of learning to give patients a voice in nursing care, and thus granting respect to residents as rights holders.

Awareness about the right to food and the capacity of developing a language about the right to food helped the students to identify challenges around the nutrition situation that arise from a structural level and that are often overlooked.^7,13,26^ In the present study, the nursing students recognized and addressed several structural challenges: in particular, the lack of professional chefs to prepare food and meals – a task that was left to the nursing students or healthcare staff; the lack of individual choice during mealtimes; the fact that some residents had to isolate themselves in their rooms due to stressful mealtimes with the other residents and inadequate routines for nutritional screening and documentation.^37,38^

Our findings also seem to indicate that adopting a human rights perspective may increase students’ awareness of ethical challenges that arise from dual loyalties, that is, conflict between nurses’ professional duties to their patients and their loyalty to an employer or other authority.^45^ The necessity that individual nurses recognize how they can achieve ‘bottom-up influence’ has become increasingly evident.^52,53^Another important finding from this study is that students participating in the coursework expressed awareness of the fact that the right to food cannot be taken for granted in their own country. HRE has been criticized for being a way to point the finger towards others for violating human rights without critical self-reflecting on their own context.^54^ A recent doctoral thesis^55^ argues that HRE is built around dichotomizations, and Norway is often presented in contrast ‘to foreign countries’ where human rights violations occur. Rights violations are thus seen as a problem outside rather than within Norway’s borders; this may make students’ own context feel less relevant even though, as noted earlier, nursing students and health professionals meet ethical challenges and rights violations on a daily basis.^45^ Further, as another study has shown, the majority of nurses experience the most frequent ethical concerns around protecting patients’ rights.^56^ This further supports the necessity of tailoring HRE to the students’ own context, making it more meaningful and relevant to their practice.

Our findings indicate that students did discuss the ethical challenges they experienced and recognized that many of these challenges could be addressed with a human rights perspective. However, as we learned from our study, students in their first clinical placement cannot be expected to change routines and break down structural barriers to promote human rights; even if they did contribute to an improvement in some instances, in most cases the challenges and rights violations continued. Residents continued to be put to bed early without being given supper, and the students were still tasked with preparing meals for the residents despite their little experience in cooking.

Thus, for the nursing students, applying their human rights competence was often limited by the structures of the institution and the healthcare service and was often beyond the nursing students’ capacity. These findings suggest that more work needs to be done to include a systems perspective in nursing care and therefore in nursing ethics education. Combining an HRBA with HRE may be one way to address this: an HRBA teaches nurses how to understand their role within the healthcare system, and an HRE is about not realizing patients’ rights in singular, heroic moments, but making meaningful changes from within the institutional system.^17^ HRE can provide a language with which to mobilize and articulate justice concerns.^16,57^

### Importance of learning about human rights through students’ relationships and experiences

The second lesson learned came with the insight that the students learned through their interactions and relationships. In our study, we found that learning took place when students talked about and reflected upon their own experiences with other students and with their supervisors, but also in their interactions with the residents.^37,38^ The emphasis on context and relationship is not new to HRE. For instance, Tibbits argues that human rights learning and accountability develop through participation and socialization.^39^ Our findings are also in line with the strong emphasis on contextual learning in the tradition of nursing education: learning in practice and clinical settings is a crucial component in nursing education.^58^ As with clinical procedures and practical patient care, knowing the theory about specific policies or human rights conventions does not make one capable of promoting human rights and advocating for social justice in practice. We found that human rights need to be contextualized for the learner. As such, HRE can benefit from being tailored to the students’ experiences and own context.^59^

In our study, the assignments required that the students discussed their own experiences, including their engagement and encounters with the residents, with a supervisor and other students. Gallagher^60^ emphasizes the importance of providing the time and space to reflect and to prioritize sensitive interactions and time over speed. The findings from our study seem to be in line with this understanding. The importance of discussing actual ethical challenges for nursing students has been acknowledged in other studies,^61,62^ as well as the need for ethics education to take into consideration the students’ experience during placement.^63^ While ethical reflection in nursing education is not uncommon,^61,63^ it appears from our study that looking at their own experiences through a human rights perspective fostered reflection and discussion, as the students shared many of the same ethical challenges. This in turn seemed to empower and, in some cases, led to action. For instance, this was the case when a patient was put to bed too early by a member of the staff and missed out on the evening meal. Some of the students discussed this with their supervisor, who brought this problem to the management.^38^ Tibbits argues that conditions that affect personal experiences can support groups to collectively develop strategies to address these problems.^39^

Hence, if the goal is to help nursing students to develop an awareness of human rights, the curriculum ought to include opportunities for discussion and reflections around ethical challenges and rights violations. Study findings also show how the students’ learning occurred through exchanging their experiences with others.^38^ An important contribution in this regard comes from the theory of learning in communities of practice (CoP).^64^ The idea that learning is a social process happening in CoP is important in nursing education.^65^ In a previous publication from the same study, we saw the importance of understanding learning as something that finds place in a community of practice, centrally involving interaction with both expert practitioners and peers.^38^ This finding indicates the need to enrich HRE with other perspectives, as HRE has been criticized for being decontextualized.^39,59^ The theory of CoP can supplement HRE by emphasizing how learning happens through socialization.^64^

### The benefits of combining the development of ethical competence and a human rights perspective

The third lesson learned was that there is a benefit of combining the students’ development of ethical competence with a human rights perspective. The students in the study reported facing several ethical challenges related to food and meals in their placement. One important dimension of nutritional care that the students brought to the table centred around the ethical challenges they experienced in their everyday care for the residents. For example, some of the students described the challenges of navigating the fine line between coercion and motivation – in particular when a resident who was malnourished did not want to eat.

Nursing education has a long tradition for supporting students in developing ethical competence. Ethical competence has been referred to by Cannaerts et al.^66^ following Gallagher definition^67^ as ‘the possession of ethical knowledge next to the ability to “see” what a situation presents (ethical perception); to reflect critically about what nurses know, are, and do (ethical reflection); to bring out the ethical practice (ethical behaviour), and to “be” ethical’. However, the nursing literature on ethics in day-to-day care concerning food and meals is sparse, compared to more existential issues, such as the ethics and challenges regarding artificial fluids at end-of-life or for patients with severe dementia.^48^

Introducing a human rights perspective seemed to provide the students with an approach that could help them deal with these ethical challenges. Applying the concepts from a HRBA, the students emphasized the values of social justice, non-discrimination, dignity and participation when determining what the situation was about, critically reflecting upon it and deciding what to do. The human rights perspective emphasized the issue of food as an *ethical* issue in nursing care, and not merely an issue of basic, physiological needs. The students discussed how they had applied these principles in a daily practice of caring, such as when they supported the residents’ right to eat by themselves and thus emphasized their autonomy, participation and dignity. The students explored what it can mean when a patient turns down an offer of food, and the balance between facilitating, motivating, tempting and forcing in mealtime settings. Students also gave several examples of instances when they promoted the right to food: understanding food as a tradition by respecting the residents’ food-related wishes or when they supported the residents to choose with whom they wanted to share mealtimes and sit together with.

A human rights perspective typically places more emphasis on structural barriers to under- and malnutrition^68^ than the traditional emphasis on ethics of care, where the nurse–patient relationship is at the core.^69^ Including a structural perspective in nursing care has received limited attention in nursing ethics education.^70^ A human rights perspective may be one way to bridge this gap, as this perspective looks beyond the individual patient–provider relationship to examine structural challenges, systemic issues and state responsibility.^17^ A human rights perspective places patients at the centre and focuses attention on universal and inherent ethical values such as dignity and participation.^16^ This means that a human rights perspective can further complement bioethics in identifying systemic issues – providing approaches and norms that are rooted in national and international law^16^ – regarding nutritional care.

## Strengths and limitations

The rich data material based on the shared experiences from the nursing students represents the strengths of this study. Another strength is that conducting the focus group interviews before, during and after the students’ placement in round 1 of the coursework, and shortly after the students’ clinical placement in round 2, may have led to more reflections from students on their real-life experiences. Moreover, the students’ familiarity with the theme ‘the right to food’ and with the other focus group participants may have allowed them to reflect more deeply on certain aspects.

One limitation of the study is that the first author taught and developed the 2-day module, which could have influenced students’ responses in the focus groups and contributed to bias. To reduce bias, the students were informed before the focus group interviews that participation in the study would not affect their evaluation and there were no correct or incorrect answers as it was important to hear their experiences and perspectives. Additional limitations were that the participants were all female, and their placements varied from the first to the second round, both of which may have influenced their answers. Finally, attendance at the lectures was voluntary, and not all the students included in the study participated in the 2-day module.

## Conclusion

The study indicates that a human rights perspective can contribute to nursing education by providing students with an understanding of nutritional care going beyond food as a basic physical need. Introducing a human rights perspective challenged the narrative of ‘vulnerable and malnourished patients’ in nursing homes. The ability to draw on the language of human rights also seemed to empower the students’ and support their navigation when they experienced ethical challenges and rights violations during placement. As the importance of including a structural perspective in nursing care has received limited attention in nursing ethics education,^70^ an HRBA can contribute to addressing this limitation.

The study also provides valuable contributions on how to introduce HRE in nursing education. Our study shows that nursing students learned about human rights through their relationships and experiences. Thus, HRE can be strengthened by being contextualized into the students’ own context for learning and the relationships they experience.^49,59^ Incorporating human rights in nursing education can support nursing students and nurses in recognizing and addressing ethical and structural challenges and promote the right to food among older adults in institutions.
